# Correction: Sex differences in chronic kidney disease awareness among US adults, 1999 to 2018

**DOI:** 10.1371/journal.pone.0273020

**Published:** 2022-08-09

**Authors:** The PLOS ONE Staff

The twelfth author’s name is spelled incorrectly. The correct name is: Allison Tong.

The publisher apologizes for the error.
