# Supplementary material for: Sex differences in chronic kidney disease awareness among US adults, 1999 to 2018
Source: PLoS One. 2020 Dec 18;15(12):e0243431. doi: 10.1371/journal.pone.0243431 (PMC7748269; doi:10.1371/journal.pone.0243431)
Supplement: S1 Table — Study sample characteristics of CKD positive and CKD negative NHANES 1999 to 2018 participants who took part in the medical examinations; number of participants and weighted percentages. (DOCX) [file pone.0243431.s001.docx]

|  | CKD | No CKD |
| --- | --- | --- |
| Sex |  |  |
| Women | 2325 (59.1) | 25040 (51.5) |
| Men | 2086 (40.9) | 23245 (48.5) |
| Race/Ethnicity |  |  |
| Caucasian | 2762 (80.8) | 20445 (67.2) |
| African American | 827 (9.3) | 10284 (11.4) |
| Mexican American | 378 (2.8) | 8829 (8.5) |
| Other | 444 (7.1) | 8727 (12.9) |
| Age Mean (SD) | 73.0 (10.2) | 47.4 (17.3) |
| [20,49] | 142 (4.6) | 26878 (60.9) |
| [50,64] | 646 (18.7) | 12070 (25.2) |
| [65,79] | 1932 (44.5) | 7344 (11.4) |
| 80+ | 1691 (32.1) | 1993 (2.5) |
| Diabetes |  |  |
| No | 3142 (74.7) | 43241 (92.3) |
| Yes | 1269 (25.3) | 5044 (7.7) |
| Hypertension |  |  |
| No | 868 (22.0) | 29997 (66.5) |
| Yes | 3543 (78.0) | 18288 (33.5) |
| BMI Mean (SD) | 29.4 (6.5) | 28.9 (6.8) |
| N-Miss | 201 | 872 |
| <25 | 1048 (24.4) | 14334 (31.7) |
| Overweight | 1523 (35.8) | 15937 (33.1) |
| Obese | 1649 (39.8) | 17142 (35.1) |
| Smoker |  |  |
| N-Miss | 2227 | 28138 |
| No | 1801 (82.2) | 11168 (55.1) |
| Yes | 383 (17.8) | 8979 (44.9) |
| Income |  |  |
| N-Miss | 169 | 1938 |
| >20.000 | 2920 (75.2) | 37039 (86.1) |
| <20.000 | 1322 (24.8) | 9308 (13.9) |
| Education |  |  |
| High School or higher | 2908 (74.6) | 35469 (83.0) |
| less than High School | 1503 (25.4) | 12816 (17.0) |
| Health Insurance |  |  |
| No | 201 (4.3) | 10980 (18.9) |
| Yes | 4210 (95.7) | 37305 (81.1) |
| Healthcare Visits |  |  |
| N-Miss | 14 | 37 |
| 0 | 140 (2.9) | 8429 (16.8) |
| 1-3 | 1420 (33.5) | 21991 (47.7) |
| 4-12 | 1713 (39.2) | 11541 (23.3) |
| >12 | 1124 (24.4) | 6287 (12.2) |
