# Supplementary material for: Sex differences in chronic kidney disease awareness among US adults, 1999 to 2018
Source: PLoS One. 2020 Dec 18;15(12):e0243431. doi: 10.1371/journal.pone.0243431 (PMC7748269; doi:10.1371/journal.pone.0243431)
Supplement: S2 Table — Study sample characteristics of CKD stage G4 and G5 and CKD-negative NHANES 1999 to 2018 participants who took part in the medical examinations; number of participants and weighted percentages. (DOCX) [file pone.0243431.s002.docx]

|  | CKD Stage G4&G5 | no CKD |
| --- | --- | --- |
| Sex |  |  |
| Female | 228 (61.4) | 25040 (51.5) |
| Male | 176 (38.6) | 23245 (48.5) |
| Race/Ethnicity |  |  |
| Caucasian | 192 (65.3) | 20445 (67.2) |
| African American | 109 (17.6) | 10284 (11.4) |
| Mexican American | 56 (6.4) | 8829 (8.5) |
| Other | 47 (10.7) | 8727 (12.9) |
| Age Mean (SD) | 71.6 (12.5) | 47.4 (17.3) |
| [20,49] | 29 (9.2) | 26878 (60.9) |
| [50,64] | 59 (12.7) | 12070 (25.2) |
| [65,79] | 159 (38.9) | 7344 (11.4) |
| 80+ | 157 (39.3) | 1993 (2.5) |
| Diabetes |  |  |
| No | 230 (58.4) | 43241 (92.3) |
| Yes | 174 (41.6) | 5044 (7.7) |
| Hypertension |  |  |
| No | 41 (10.9) | 29997 (66.5) |
| Yes | 363 (89.1) | 18288 (33.5) |
| BMI Mean (SD) | 30.0 (6.8) | 28.9 (6.8) |
| N-Miss | 24 | 872 |
| <25 | 90 (23.4) | 14334 (31.7) |
| overweight | 126 (32.2) | 15937 (33.1) |
| obese | 164 (44.4) | 17142 (35.1) |
| Smoker |  |  |
| N-Miss | 191 | 28138 |
| No | 173 (76.7) | 11168 (55.1) |
| Yes | 40 (23.3) | 8979 (44.9) |
| Income |  |  |
| N-Miss | 18 | 1938 |
| >20.000 | 260 (71.5) | 37039 (86.1) |
| <20.000 | 126 (28.5) | 9308 (13.9) |
| Education |  |  |
| High School or higher | 237 (65.5) | 35469 (83.0) |
| less than High School | 167 (34.5) | 12816 (17.0) |
| Health Insurance |  |  |
| No | 16 (3.0) | 10980 (18.9) |
| Yes | 388 (97.0) | 37305 (81.1) |
| Healthcare Visits |  |  |
| N-Miss | 3 | 37 |
| 0 | 8 (1.2) | 8429 (16.8) |
| 1-3 | 88 (20.2) | 21991 (47.7) |
| 4-12 | 149 (35.2) | 11541 (23.3) |
| >12 | 156 (43.4) | 6287 (12.2) |
