# Supplementary material for: Sex differences in chronic kidney disease awareness among US adults, 1999 to 2018
Source: PLoS One. 2020 Dec 18;15(12):e0243431. doi: 10.1371/journal.pone.0243431 (PMC7748269; doi:10.1371/journal.pone.0243431)
Supplement: S3 Table — This table refers to Fig 2, providing adjusted CKD awareness odds ratios (with 95% confidence intervals) for all (left), Caucasian (middle) and African American (right) study participants; odds ratios were adjusted for all other characteristics shown, odds ratios for all participants were further adjusted for race/ethnicity; within each model, women with one of the characteristics constituted the reference group. (DOCX) [file pone.0243431.s003.docx]

|  | All | | Caucasian | | African  American | |
| --- | --- | --- | --- | --- | --- | --- |
| Model | Men | Women | Men | Women | Men | Women |
| Period |  |  |  |  |  |  |
| 1999-2002 | 2.60 (1.34,5.04) | 1.00 (ref) | 3.72 (1.43,9.68) | 1.00 (ref) | 0.82 (0.27,2.47) | 1.00 (ref) |
| 2003-2006 | 1.98 (1.04,3.78) | 1.32 (0.68,2.55) | 2.79 (1.11,7.03) | 1.95 (0.76,5.00) | 1.23 (0.44,3.41) | 0.57 (0.19,1.75) |
| 2007-2010 | 2.20 (1.19,4.04) | 1.12 (0.61,2.08) | 3.10 (1.27,7.60) | 1.47 (0.59,3.69) | 0.73 (0.25,2.14) | 0.51 (0.20,1.31) |
| 2011-2014 | 2.16 (1.15,4.06) | 1.56 (0.82,2.95) | 2.99 (1.17,7.67) | 2.05 (0.79,5.33) | 1.26 (0.49,3.22) | 1.12 (0.44,2.83) |
| 2015-2018 | 3.84 (2.08,7.09) | 2.84 (1.55,5.22) | 5.01 (2.01,12.53) | 4.25 (1.72,10.48) | 1.96 (0.74,5.24) | 1.40 (0.57,3.44) |
| CKD Stage |  |  |  |  |  |  |
| G3 | 1.56 (1.18,2.06) | 1.00 (ref) | 1.51 (1.07,2.15) | 1.00 (ref) | 1.53 (0.93,2.50) | 1.00 (ref) |
| G4 | 12.45 (7.65,20.28) | 6.51 (4.23,10.01) | 13.30 (7.35,24.04) | 5.18 (2.84,9.45) | 4.04 (1.50,10.84) | 5.88 (2.80,12.33) |
| G5 | 62.48 (21.30,183.30) | 25.30 (9.36,68.38) | 3.2x10^7^ (1.3x10^7^,7.7x10^7^) | 64.76 (10.26,408.91) | 8.76 (2.92,26.31) | 22.57 (4.82,105.70) |
| Age |  |  |  |  |  |  |
| [20,49] | 2.17 (0.75,6.30) | 1.00 (ref) | 3.40 (0.52,22.16) | 1.00 (ref) | 0.92 (0.18,4.61) | 1.00 (ref) |
| [50,64] | 0.74 (0.30,1.80) | 0.72 (0.29,1.79) | 0.67 (0.14,3.24) | 0.95 (0.20,4.54) | 0.68 (0.19,2.35) | 0.35 (0.10,1.21) |
| [65,79] | 0.78 (0.34,1.80) | 0.40 (0.17,0.92) | 1.01 (0.23,4.41) | 0.44 (0.10,1.98) | 0.21 (0.06,0.67) | 0.19 (0.06,0.62) |
| 80+ | 0.56 (0.24,1.31) | 0.37 (0.16,0.86) | 0.67 (0.15,2.98) | 0.46 (0.10,2.04) | 0.20 (0.05,0.79) | 0.19 (0.05,0.64) |
| Diabetes Mellitus (DM) |  |  |  |  |  |  |
| No | 1.58 (1.15,2.17) | 1.00 (ref) | 1.50 (1.01,2.22) | 1.00 (ref) | 1.57 (0.84,2.92) | 1.00 (ref) |
| Yes | 3.45 (2.36,5.07) | 2.07 (1.43,2.99) | 3.64 (2.25,5.89) | 1.94 (1.18,3.18) | 2.28 (1.17,4.47) | 2.31 (1.31,4.08) |
| Hypertension (HT) |  |  |  |  |  |  |
| No | 1.37 (0.67,2.82) | 1.00 (ref) | 1.53 (0.64,3.67) | 1.00 (ref) | 0.67 (0.12,3.70) | 1.00 (ref) |
| Yes | 2.52 (1.39,4.57) | 1.52 (0.85,2.74) | 2.66 (1.24,5.70) | 1.62 (0.76,3.42) | 3.26 (1.01,10.53) | 2.45 (0.76,7.87) |
| BMI |  |  |  |  |  |  |
| <25 | 2.26 (1.32,3.88) | 1.00 (ref) | 2.82 (1.42,5.62) | 1.00 (ref) | 0.99 (0.25,3.88) | 1.00 (ref) |
| overweight | 2.77 (1.72,4.44) | 1.59 (0.94,2.68) | 3.31 (1.81,6.08) | 1.62 (0.81,3.25) | 2.97 (1.04,8.51) | 4.18 (1.46,11.94) |
| obese | 3.04 (1.90,4.87) | 2.20 (1.39,3.49) | 3.47 (1.88,6.43) | 2.88 (1.58,5.25) | 3.04 (1.09,8.44) | 1.49 (0.55,3.99) |
