# Supplementary material for: Sex differences in chronic kidney disease awareness among US adults, 1999 to 2018
Source: PLoS One. 2020 Dec 18;15(12):e0243431. doi: 10.1371/journal.pone.0243431 (PMC7748269; doi:10.1371/journal.pone.0243431)
Supplement: Attachment — Submitted filename: Response to Reviewers.docx [file pone.0243431.s004.docx]

To the Editors of PLOS One

11. November 2020

Dear Dr. Kronenberg,

we thank you for the opportunity to submit a revised version of our manuscript ***“Sex Differences in Chronic Kidney Disease Awareness among US Adults, 1999 to 2018”*** . In the revised version of our manuscript, we have addressed the comments raised by the reviewers and have also ensured that the publication criteria of the Journal would be fulfilled. Our detailed rebuttal letter is attached here below.

As concerns funding, we declare the following:

“The funders had no role in study design, data collection and analysis, decision to publish, or preparation of the manuscript. There was no additional external funding received for this study."

As concerns data availability, we declare the following:

“The present study is based on NHANES data, which are publicly available at <https://wwwn.cdc.gov/nchs/nhanes/Default.aspx> . The code of our statistical analyses has been uploaded to the Protocols.io website and can be accessed using the following link: *https://protocols.io/file/cp9hbg8yp.r*.

The present manuscript, as previously stated, is an important piece in the puzzle surrounding the discrepancy between higher CKD prevalence among women and lower dialysis rates. We would therefore appreciate timely publication in *PLOS One*.

With kind regards,

Manfred Hecking, MD, PhD
Eva Schernhammer, MD, DrPH, MPH, MSc (corresponding author)

**Rebuttal Letter**

Journal Requirements:

1. Please ensure that your manuscript meets PLOS ONE's style requirements, including those for file naming. The PLOS ONE style templates can be found at

<https://journals.plos.org/plosone/s/file?id=wjVg/PLOSOne_formatting_sample_main_body.pdf> and

<https://journals.plos.org/plosone/s/file?id=ba62/PLOSOne_formatting_sample_title_authors_affiliations.pdf>

*🡪 Our Reply: PLOS ONE’s style requirements have been adopted and are now fulfilled in the revised manuscript.*

2. Please provide an amended statement that declares *all* the funding or sources of support (whether external or internal to your organization) received during this study, as detailed online in our guide for authors at [http://journals.plos.org/plosone/s/submit-now](about:blank).  Please also include the statement "There was no additional external funding received for this study." in your updated Funding Statement.

Please include your amended Funding Statement within your cover letter. We will change the online submission form on your behalf.

*🡪 Our Reply: We have included our amended Funding Statement into our cover letter here above, as requested. Thank you for changing the online submission form on our behalf.*

3. We note that you have stated that you will provide repository information for your data at acceptance. Should your manuscript be accepted for publication, we will hold it until you provide the relevant accession numbers or DOIs necessary to access your data. If you wish to make changes to your Data Availability statement, please describe these changes in your cover letter and we will update your Data Availability statement to reflect the information you provide.

*🡪 Our Reply: Our manuscript is based on NHANES data, which are accessible through the NHANES website. The code of our statistical analyses has been uploaded to the Protocols.io website and can be accessed using the following link: https://protocols.io/file/cp9hbg8yp.r . We have included a full Data Availability statement into our cover letter here above. Thank you for changing the online submission form on our behalf.*

4. We note that you have included the phrase "data not shown" in your manuscript. Unfortunately, this does not meet our data sharing requirements. PLOS does not permit references to inaccessible data. We require that authors provide all relevant data within the paper, Supporting Information files, or in an acceptable, public repository. Please add a citation to support this phrase or upload the data that corresponds with these findings to a stable repository (such as Figshare or Dryad) and provide and URLs, DOIs, or accession numbers that may be used to access these data. Or, if the data are not a core part of the research being presented in your study, we ask that you remove the phrase that refers to these data.

*🡪 Our Reply: We understand your request, have included the missing data into our revised Table 1 and have removed the respective phrase (‘data not shown’)*

5. Please remove your figures from within your manuscript file, leaving only the individual TIFF/EPS image files, uploaded separately.  These will be automatically included in the reviewers' PDF.

*🡪 Our Reply: The figures have been removed from within our manuscript.*

6. Please include captions for your Supporting Information files at the end of your manuscript, and update any in-text citations to match accordingly. Please see our Supporting Information guidelines for more information: [http://journals.plos.org/plosone/s/supporting-information](about:blank).

*🡪 Our Reply: The captions for the Supporting Information files were included at the end of the manuscript. We hope that this procedure fulfills your request.*

7. PLOS requires an ORCID iD for the corresponding author in Editorial Manager on papers submitted after December 6th, 2016. Please ensure that you have an ORCID iD and that it is validated in Editorial Manager. To do this, go to 'Update my Information' (in the upper left-hand corner of the main menu), and click on the Fetch/Validate link next to the ORCID field. This will take you to the ORCID site and allow you to create a new iD or authenticate a pre-existing iD in Editorial Manager. Please see the following video for instructions on linking an ORCID iD to your Editorial Manager account: <https://www.youtube.com/watch?v=_xcclfuvtxQ>

*🡪 Our Reply: Eva Schernhammer is the corresponding author of this manuscript and has updated her information, so that her ORCID iD (0000-0002-4337-9415) is now included.*

8. Please include your tables as part of your main manuscript and remove the individual files. ** Please note that supplementary tables (should remain/ be uploaded) as separate "supporting information" files **.

*🡪 Our Reply: We have included the tables as part of our main manuscript, as requested.*

Reviewers' comments

Reviewer #1: The authors examine NHANES data to conclude that CKD awareness is lower in women than in men and that the gap between the sexes is narrowing due to greater reliance on eGFR, replacing serum creatinine, as a measure of renal function

Major

1. NHANES does not provide data regarding access to healthcare. There are no data to assess the frequency which women versus men visit PCPs or are referred for consultation to a nephrologist. Prior publications indicate that women are referred to nephrologists less frequently than men and data from the USRDS suggests that women undergo fewer serum creatinine measurements than men. Less contact with PCPs and nephrologists and fewer serum creatinine measurements may contribute to lesser awareness of CKD. The authors should include this limitation in their discussion.

*🡪 Our Reply: We thank the reviewer for this insightful comment. We absolutely agree and have included this thought into the Limitations passage of the revised Discussion. However, we could not identify the references for sex differences in referral and for fewer serum creatinine measurements among women.* ***We would be very grateful if you could send these references during your next round of corrections.*** *The revised Limitations paragraph (so far) now reads as follows:*

*“As regards healthcare access as an explanatory factor for the present study findings, our analysis, regrettably, is limited by the fact that NHANES does not provide these data. Thus we were unable to assess the frequency by which women versus men visit primary care physicians or are referred for consultation to a nephrologist. Women might be referred to nephrologists less frequently than men, as men have been shown to predominate in several CKD cohorts, such as from the US (1), Canada (2), Japan (3), Germany (4), China (5) and France (6). Less contact with nephrologists may therefore contribute to lesser awareness of CKD.”*

Minor

1. The authors point to a large body of literature that indicates that CKD is less prevalent in men than in women. In this context, they should discuss limitations of these data including (1) differences in bias between the sexes in the GFR estimating equations and (2) controversy regarding the need for sex/age specific GFR cut offs and UACR cutoffs to define CKD.

*🡪 Our Reply: We thank the reviewer for this additional comment. In our Discussion paragraph, we had previously addressed the issue that age-specific cut-offs for CKD stages might perhaps be required, (as suggested for instance by Glassock et al. (7)), but we have now also incorporated the idea, as well as the potential miscalculation of the eGFR among women (8) in the revised Introduction.*

2. In the Introduction section the authors point out that women start dialysis at a lower GFR than men. However they should also point out that this observation does not account for the difference in bias between the sexes in the GFR estimating equations which is equivalent to the difference in the GFR at the start of dialysis in men vs. women.

*🡪 Our Reply: This is an interesting remark. For your information, we have recently started analyzing CKDopps data, where the difference in eGFR at dialysis initiation in men versus women can also be observed, although the difference was less pronounced in the CKDopps dataset than in the cited references. This finding has made us doubt the idea that late KRT initiation can explain much of the sex difference in dialysis initiation. Instead, more women than men might be remaining in the CKD clinics with very low eGFRs, without ever initiating dialysis. In other words, “hanging out there with low eGFRs.” (Of note, the individuals from CKDopps were only nephrologist-referred patients.)*

*With the point that you are making here above, we believe you are suggesting something else, namely that whoever chooses to initiate dialysis in men and women (most likely the treating physician, sometimes the patient [but, as data from Sara Davison indicate: most often the doctor alone (9)]) in any case this individual in charge might be aware of the fact that the formulas for eGFR overestimates the degree of kidney failure in women, and that this awareness of the eGFR miscalculation would lead the doctor to conclude: “Starting dialysis in this female patient (by eGFR) is less necessary because the eGFR is falsely too low, anyways (because the patient is a female).” We have doubts that this might be the case, as we believe the miscalculation of eGFR, if anything, would lead to an earlier dialysis start among women. Please feel free to let us know your thoughts on this issue, but we believe that the point you are suggesting might be a bit hard to make.*

3. The authors indicate that serum creatinine was corrected and cite several references -- NHANES Data Documentation, Codebook, and Frequencies. Since standardization of creatinine assays is an important issue in evaluating their data and conclusions, the authors should describe in some detail how the correction was performed.

*🡪 Our Reply: Thank you for this comment. We have added the correction factors for serum creatinine from the NHANES analytical guidelines to the revised version of the manuscript (Methods section, subheading ”CKD staging”).*

4. The authors may wish to speculate as to why it is that Caucasian women drive the increase in CKD awareness over time.

*🡪 Our Reply: Thank you, but this is a tough call. Perhaps better educational (and socioeconomic) status among Caucasians might be an underlying causal factor, but African Americans were generally more aware of CKD than Caucasians, which could be a community issue (for example, extended African American families may be quite used to some relatives attending dialysis). We have tried to add this speculation to the manuscript (first paragraph of the Discussion), as follows below:*

*”The rise in CKD awareness levels in the US population was to a large part driven by growing CKD awareness rates in Caucasian women (from 8.5% in the first to 18.0% in the last 4-year period considered), while the improvement in the sex difference among African Americans was markedly smaller. Although this finding might be a consequence of differing education levels, African Americans had generally higher awareness rates than Caucasians, perhaps related to better communication of the risk associated with CKD within the African American community, which is however a purely speculative thought.”*

*Please let us know if you think that this statement should be revised further.*

5. The authors may wish to point out the weakness of data from the USRDS that seem to contradict their conclusions. USRDS data show that women are as likely as men to receive pre-ESRD care from a nephrologist, strongly suggesting equivalent CKD awareness in men and women in late stage CKD. However, these data are seriously limited since they only apply to individuals who ultimately start dialysis.

*🡪 Our Reply: Thank you for this comment. As you said, in the 2019 USRDS Annual Data Report (10), we identified duration of pre-ESRD nephrology care (>12 months and other categories) in Table 11a, which was similar among men and women. We agree with you that these data are limited by the fact that they refer only to patients who ultimately start dialysis. Perhaps related to this issue, we were surprised to observe that even among those with eGFR <15 mL/min/1.73m^2^, not all individuals reported having been told they had weak or failing kidneys (with a consistent sex difference [i.e., higher awareness among men] as in earlier CKD stages). It would be interesting to study the degree of CKD unawareness even in CKD clinics. (A personal note: we have experienced that patients had some CKD clinic visits, but when a medical school student asked them why they were coming, they did not know.) We have added your thought to the Discussion; thank you again.*

6. In general the paper is clear, well written and easy to understand. However, on several occasions the authors misuse idioms that obfuscate the meaning of sentences. For example: "consecutively declining" and "tilt towards". (Unfortunately the pages are not numbered so I cannot identify these phrases by page and line number).

*🡪 Our Reply: Thank you for these additional corrections. The word choice was improved here, and we read through the manuscript several times further, correcting additional spots where the language might have been problematic.*

Reviewer #2: Hödlmoser et al. investigated the sex difference in the awareness of CKD using data from US NHANES. Overall, the awareness was low <22.5%. The authors observed higher awareness in men than in women, but the sex-difference became smaller overtime. The sex difference was more evident in whites than in blacks. The study question is important and the manuscript is well written overall. Nonetheless, I have several suggestions and comments as summarized below.

Major
1. The definition of CKD.
a. "CKD is characterized by a gradual loss of kidney function," Kidney damage is a part of CKD, which may not be accompanied by reduced kidney function. This sentence thus should be rephrased.

*🡪 Our Reply: Thank you for your comment. We checked the definition of CKD from various sources and did identify “gradual decrease in kidney function” as a common theme. However, we now rephrased the sentence as follows: “CKD occurs in consequence of kidney damage and is usually characterized by a gradual loss of kidney function, […].” We hope you may agree with this approach.*

b. "we restricted our study to CKD stages 3 to 5, which are based solely on eGFR" Please specify that these are KDIGO CKD G stages. This comment applies to the entire manuscript.

*🡪 Our Reply: Absolutely, thank you very much! This was changed throughout the manuscript.*

2. Not sure whether the authors reported weighted results for the descriptive statistics in the section of "Characteristics of the study sample". Please specify. If the authors reported unweighted estimates, they should report the weighted results instead.

*🡪 Our Reply: Thank you for asking. The Methods section and the Tables made clear that these are weighted percentages. For clarity, we inserted an additional remark into the respective paragraph of the Results section which you were mentioning.*

3. Figure 2 was blank in the PDF.

4. Figure 2 may be trying to show this, but it seems important to show an analysis restricted to participants with diabetes, hypertension, or both.

*🡪 Our Reply: We sincerely apologize. In principle, this issue (of the missing figure) was picked up and corrected by the corresponding author, but an unedited version of the PDF seems to have been sent out. For your information, we have attached Figure 2 here below:*

*As you can see, it would not make sense to restrict the analysis shown in this Figure to patients with hypertension, diabetes or both, as the data in Figure 2 were already analyzed by presence (versus absence) of diabetes and presence (versus absence) of hypertension. However, we thought that your request could be fulfilled best by restricting Figure 3 to patients with diabetes, hypertension or both.*

*Figure 3, all patients:*

*Figure 3, sensitivity analysis 1 (diabetes):*

*Figure 3, sensitivity analysis 2 (hypertension):*

*Figure 3, sensitivity analysis 3 (diabetes + hypertension):*

*As already shown in Figure 2 (which you were unable to access), individuals with diabetes and to a lesser degree those with hypertension (among the subgroup in NHANES who all had CKD) were more aware of their “weak or failing kidneys”. However, the sex difference in CKD awareness remained quite consistent. We decided not to place particular emphasis on this detail, but would be interested in hearing your additional thoughts.*

Minor
Abstract
1. Please specify that sampling weights were taken into account in the Abstract.

*🡪 Our Reply: Thank you for your remark, we included the information, that all percentages are survey-weighted into the Abstract.*

Methods
2. "[20 to 50 years], [50 to 65 years], [65 to 80 years] and 80+ years" Not clear which category included 50, 65, and 80 years old.

*🡪 Our Reply: Thank you for pointing out that the age group labels were not clear. We rephrased the definition and relabeled all tables and figures such as to emphasize that the age groups were defined as 20-49, 50-64, 65-79 and 80+ years.*

Results
3. "most of the study participants (92.7%) had CKD stage 3" This seems misleading. I believe the authors meant "most of the study participants with CKD had G stage 3"

*🡪 Our Reply: Thank you! The respective sentence was corrected.*

4. "Due to the low number of participants with CKD stage 5, the volatility and decrease of awareness in both sexes should be considered with caution." This is not a result and thus should be moved to Discussion.

*🡪 Our Reply: Thank you! This sentence was moved to the Limitations paragraph of the Discussion, where we believe it fits best.*

5. "Figure 4 s" s should not be in bold.

*🡪 Our Reply: This was corrected, thank you.*

Discussion
6. The authors should discuss higher awareness in blacks than in whites.

*🡪 Our Reply: We agree with you and have addressed this issue in the first paragraph of the revised Discussion.*

7. "Chronic kidney disease is defined by abnormalities of kidney function," The defined abbreviation of CKD should be used.

*🡪 Our Reply: This was corrected, thank you.*

Reviewer #3: The review is too difficult to read. The main problem is due to a different diseases that can lead to CKD. For example the glomerulonephrithis should be considered. Also CKD should be diagnosed also considered urine output, proteinuria, ecc

*🡪 Our Reply: Thank you for taking the time to review our manuscript. We hope that the changes we have made have improved the manuscript flow and understandability. As regards your concern, that sex differences in CKD awareness may be driven by the underlying disease, we hope that the sensitivity analysis provided in response to Reviewer 2 may also satisfy your concern. (Your question may have to do with the fact that Figure 2, unfortunately was not legible). Briefly, individuals with diabetes and to a lesser degree those with hypertension (among the subgroup in NHANES who all had CKD) were more aware of their “weak or failing kidneys”. We did not restrict to patients with glomerulonephritis, as we were unable to retrieve this information from the NHANES dataset.*

*As regards your concern that CKD should be diagnosed by considering proteinuria, we fully agree, but as mentioned in the manuscript, data on proteinuria are not solid enough in NHANES to really be considered, which is why we kept the CKD stage definition by eGFR, in analogy with all previous studies. We hope that you may agree with this approach. As regards urine output, we agree with the importance of analyzing oliguria and anuria, especially when considering dialysis initiation, but this information is also not captured in NHANES.*

*References*

1.	Lash JP, Go AS, Appel LJ, He J, Ojo A, Rahman M, et al. Chronic Renal Insufficiency Cohort (CRIC) Study: baseline characteristics and associations with kidney function. Clinical journal of the American Society of Nephrology : CJASN. 2009;4(8):1302-11.

2.	Levin A, Rigatto C, Brendan B, Madore F, Muirhead N, Holmes D, et al. Cohort profile: Canadian study of prediction of death, dialysis and interim cardiovascular events (CanPREDDICT). BMC Nephrol. 2013;14:121.

3.	Imai E, Matsuo S, Makino H, Watanabe T, Akizawa T, Nitta K, et al. Chronic Kidney Disease Japan Cohort study: baseline characteristics and factors associated with causative diseases and renal function. Clin Exp Nephrol. 2010;14(6):558-70.

4.	Titze S, Schmid M, Kottgen A, Busch M, Floege J, Wanner C, et al. Disease burden and risk profile in referred patients with moderate chronic kidney disease: composition of the German Chronic Kidney Disease (GCKD) cohort. Nephrology, dialysis, transplantation : official publication of the European Dialysis and Transplant Association - European Renal Association. 2015;30(3):441-51.

5.	Yuan J, Zou XR, Han SP, Cheng H, Wang L, Wang JW, et al. Prevalence and risk factors for cardiovascular disease among chronic kidney disease patients: results from the Chinese cohort study of chronic kidney disease (C-STRIDE). BMC Nephrol. 2017;18(1):23.

6.	Stengel B, Metzger M, Combe C, Jacquelinet C, Briancon S, Ayav C, et al. Risk profile, quality of life and care of patients with moderate and advanced CKD: The French CKD-REIN Cohort Study. Nephrology, dialysis, transplantation : official publication of the European Dialysis and Transplant Association - European Renal Association. 2019;34(2):277-86.

7.	Glassock R, Delanaye P, El Nahas M. An Age-Calibrated Classification of Chronic Kidney Disease. JAMA : the journal of the American Medical Association. 2015;314(6):559-60.

8.	Froissart M, Rossert J, Jacquot C, Paillard M, Houillier P. Predictive performance of the modification of diet in renal disease and Cockcroft-Gault equations for estimating renal function. Journal of the American Society of Nephrology : JASN. 2005;16(3):763-73.

9.	Davison SN. End-of-life care preferences and needs: perceptions of patients with chronic kidney disease. Clinical journal of the American Society of Nephrology : CJASN. 2010;5(2):195-204.

10.	US Renal Data System 2019 Annual Data Report: Epidemiology of Kidney Disease in the United States <https://www.usrds.org/media/2371/2019-executive-summary.pdf>. 2019.
